# Double soft-tipped guidewire facilitating the rendezvous technique for ERCP

**DOI:** 10.1055/a-2119-5002

**Published:** 2023-07-13

**Authors:** Yoshihide Kanno, Haruka Okano, Fumisato Kozakai, Kento Hosokawa, Kei Ito

**Affiliations:** Sendai City Medical Center, Sendai, Japan

The rendezvous technique has been a traditional salvage technique when transpapillary cannulation is difficult 1 2 . For the rendezvous technique, a guidewire is inserted via a percutaneously or an endosonographically created route (endoscopic submucosal resection [ESR]) into the duodenum through the papilla and then caught under duodenoscopy to ensure transpapillary access from the duodenum. After successfully approaching the duct from the duodenum along the guidewire, the wire must be exchanged or inverted to use the seeking tip to seek upward ducts.

A new double soft-tipped guidewire that has two soft angulated tips with hydrophilic coating on both sides has been recently developed (RevoWave DualMaster; Piolax Medical Devices, Inc., Yokohama, Japan) ( Fig. 1 ). Using this guidewire, the cumbersome procedures of inverting the tips or other alternative maneuvers are unnecessary ( Fig. 2 ).

**Fig. 1:**
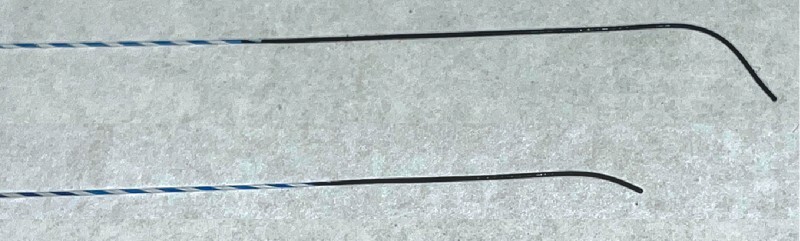
Double soft tips of the RevoWave DualMaster guidewire. One tip has a long, deep angulation, and the other has a short, light angulation. Both ends have a hydrophilic coating.

**Fig. 2:**
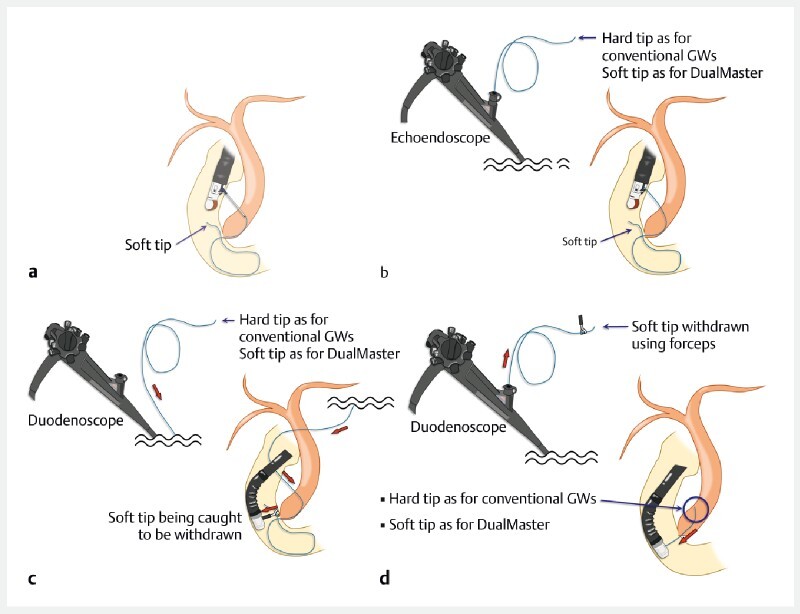
The endoscopic ultrasound (EUS)-assisted rendezvous technique. **a** After puncturing the bile duct using EUS, a guidewire is inserted into the duodenum through the papilla. **b** After the needle is withdrawn, the hard tip, which is unsuitable for seeking, is at the oral end in the case of conventional guidewires. In the case of the DualMaster, the first soft tip is located in the duodenum with the second soft tip outside the body. **c** The echoendoscope is withdrawn so that only the guidewire and a duodenoscope are inserted into the duodenum. The soft tip in the duodenum is caught and pulled using forceps through the working channel. **d** When the caught tip is sufficiently pulled, the other tip moves into the bile duct through the endosonographically created route. For conventional guidewires, the guidewire must be exchanged or inverted using a catheter because the hard tip cannot be used to negotiate upstream ducts. However, when a DualMaster is employed, negotiation and subsequent procedures can be performed without such guidewire maneuvers because the tip within the bile duct is suitable for seeking.

For an 85-year-old patient with biliary stones, the rendezvous technique was used after biliary cannulation failed owing to the papilla opening within a huge diverticulum. A DualMaster guidewire was inserted into the distal bile duct via the endosonographic route created using a 19-gauge needle ( Video 1 ). The guidewire tip, which appeared in the duodenal diverticulum through the papilla via the distal bile duct, was caught using forceps and pulled back from the working channel of the duodenoscope. After a cannula was inserted along the guidewire into the distal bile duct, the wire was pulled until the other tip disengaged from the ESR so that the tip was completely located within the bile duct. Then, the tip was advanced toward the upward bile duct to be utilized for the subsequent sphincterotomy and stone removal.

**Video 1** Rendezvous technique facilitated by the double-forefront guidewire.


Modification of the rendezvous technique using this guidewire renders guidewire exchange unnecessary, resulting in shorter procedure times and increased safety. It is a reasonable option for the rendezvous technique because the procedures are simplified.

Endoscopy_UCTN_Code_TTT_1AS_2AD

## Notes

Endoscopy E-Videos

https://eref.thieme.de/e-videos
*E-Videos* is an open access online section of the journal *Endoscopy* , reporting on interesting cases and new techniques in gastroenterological endoscopy. All papers include a high-quality video and are published with a Creative Commons CC-BY license. Endoscopy E-Videos qualify for HINARI discounts and waivers and eligibility is automatically checked during the submission process. We grant 100% waivers to articles whose corresponding authors are based in Group A countries and 50% waivers to those who are based in Group B countries as classified by Research4Life (see: https://www.research4life.org/access/eligibility/ ). This section has its own submission website at https://mc.manuscriptcentral.com/e-videos
